# Dietary content and combined training, but not daily physical activity, are associated with 6-month bone mineral changes in adolescents with obesity: A Secondary analysis of the PAC-MAnO trial

**DOI:** 10.1007/s00431-024-05659-4

**Published:** 2024-06-27

**Authors:** Antonio Videira-Silva, Inês Santos, Dalise Freaza, Mariana Gameiro, Luis B. Sardinha, Helena Fonseca

**Affiliations:** 1 Centro de Investigação Em Desporto, Educação Física, Exercício E Saúde (CIDEFES), Universidade Lusófona, Lisbon, Portugal; 2 Clínica Universitária de Pediatria, Faculdade de Medicina, Universidade de Lisboa Av. Prof. Egas Moniz, 1649-035 Lisbon, Portugal; 3 Research Centre in Physical Activity, Health and Leisure (CIFI2D), Faculty of Sport, University of Porto, Porto, Portugal; 4 Laboratório de Nutrição, Faculdade de Medicina, Centro Académico de Medicina de Lisboa, Universidade de Lisboa, Lisbon, Portugal; 5 Instituto de Saúde Ambiental (ISAMB), Faculdade de Medicina, Universidade de Lisboa, Lisbon, Portugal; 6 Centro Académico de Medicina de Lisboa, Universidade de Lisboa, Lisbon, Portugal; 7 Exercise and Health Laboratory, Faculty of Human Kinetics, University of Lisbon, Lisbon, Portugal; 8 Department of Pediatrics, Pediatric Obesity Clinic, Hospital de Santa Maria, Lisbon, Portugal

**Keywords:** Adolescents, Obesity, Diet, Physical activity, Exercise, Bone health

## Abstract

**Purpose:**

The present study aimed to explore the influence of diet and physical activity (PA) changes on bone mineral content (BMC) and density (BMD) alterations in adolescents with obesity undergoing a weight loss program.

**Methods:**

Six-month longitudinal data from 71 adolescents (aged 15.1 [± 1.6] years; 57.7% girls) with a BMI z-score of 3.03 (± 0.78), previously recruited for the PAC-MAnO trial, were analyzed using Generalized Estimation Equations for over time changes and linear regressions with BMC, BMD and BMD z-score as dependent variables, adjusting for confounders (including type of exercise- aerobic vs. combined).

**Results:**

Adjusting for confounders, changes in carbohydrate (CH) and protein content showed to positively and negatively predict BMD z-score variance, respectively (*β* = 0.44, 95%CI: 0.01, 0.04, *p* < .001); *β* = -0.57, 95%CI: -0.06, -0.03, *p* < .001), yet no associations were found between PA and bone-related parameters. Combined exercise showed better results on BMC compared to aerobic exercise (*β* = 0.09, 95%CI: 0.05 to 0.13, *p* < .001).

**Conclusions:**

Increased CH content, instead of protein, may be associated with BMD improvements in adolescents with obesity. Type of exercise may moderate the impact of PA on bone health.

**Trial registration:**

Clinicaltrials.gov NCT02941770.

**Table:** 

**What is Known** • Adolescents with obesity may be at a higher risk of osteopenia/osteoporosis • Obesity and inadequate diet and physical activity (PA) may have an adverse effect on bone metabolism	
**What is New** • Improvements in adiposity and muscle mass and increased diet carbohydrate content are associated with bone mineral density (BMD) improvements • Type of exercise (i.e., combined training vs. aerobic) may moderate the impact of PA on BMD, and calcium intake may mediate this impact

**Supplementary Information:**

The online version contains supplementary material available at 10.1007/s00431-024-05659-4.

## Introduction

Early and sustained exposure to a positive energy balance due to high consumption of energy-dense/low-quality foods and drinks [1], low levels of physical activity (PA) [2], and high levels of sedentary behaviors [3], together with a genetic component and psychosocial contributors may have driven the rising global prevalence and severity of pediatric obesity [4], anticipating the incidence of cardiometabolic comorbidities [5].

The likelihood of adolescents with obesity experiencing neuromusculoskeletal impairments, including injuries and fractures, is high [6], and may compromise their ability to function, move, and in their capacity to engage in a physically active lifestyle [7].

It has been suggested that adolescents with overweight/obesity have higher bone mineral content (BMC) and bone mineral density (BMD) compared to those with healthy weight [8]. However, other studies pointed to obesity as deleterious for bone health [9, 10], with adipokines playing a key role [11]. Moreover, obesity’s negative impact on bone health seems to be worsened by the presence of metabolic syndrome (MS) [12]. Obesity may eventually compromise peak bone mass accrual, impairing skeletal development during adolescence, increasing osteoporotic risk later in life [13].

The impact of obesity and MS on bone health, is not fully understood, with no consensus on how obesity affects BMC and BMD in adolescents [9, 12]. Environmental, behavioral, and genetic factors influence this complex relationship. Although 60–80% of peak bone mass is genetically determined [14], diet and PA behaviors (especially calcium and vitamin D intake and weight-bearing/muscle-strengthening activities) [15, 16] may account for the remaining 20- 40%.

Emerging evidence linking obesity to sub-optimal bone health in adolescents has been translated into clinical practice [17]. Yet, further research is needed in the context of weight loss programs, which, once focusing on total energy intake (TEI) restriction, may compromise calcium and vitamin D intake, consequently impacting bone health [18].

Few longitudinal studies exist on the influence of diet and PA changes on BMC and BMD in adolescents with obesity following a specialized weight management program. Thus, this study aimed to explore the influence of diet and PA changes on BMC and BMD alterations in adolescents with obesity undergoing a weight loss program, while controlling for potential confounders.

## Materials and Methods

Study design and outcomes are reported according to CONSORT-Outcomes 2022 Extension [19].

### Trial design

Relevant data from the PAC-MAnO trial (Clinicaltrials.gov NCT02941770) were analyzed in this study [20]. In brief, the PAC-MAnO trial consisted of a 3-arm non-randomized controlled trial testing the efficacy of a PA consultation-only (EGI) and a PA consultation plus weekly supervised physical exercise sessions (EGII), compared to standard care (CG) in the treatment of obesity in adolescents over 6 months, with a 6-month follow-up [20, 21]. Additional information regarding EGII exercise sessions can be found elsewhere [22].

The trial was approved by the Ethics Committee of the Faculty of Medicine of the University of Lisbon, Portugal (271/2016) and is under the 1964 Helsinki Declaration and its later amendments or comparable ethical standards.

### Participants

The PAC-MAnO enrolled 165 adolescents (12–17 years old) with overweight or obesity (BMI ≥ 85th percentile [23]) attending for the first time the Pediatric Obesity Clinic of Hospital de Santa Maria, Lisbon, a Public Central Hospital (CG, *n* = 55; EGI *n* = 55; EGII *n* = 55) [24]. Exclusion criteria included: (i) major non-obesity related pathologies, (ii) inability to perform regular PA, (iii) mental disorders, (iv) smoking, (v) prescriptions affecting weight or bone metabolism. All participants and caregivers provided informed assent/consent.

Of the 165 adolescents recruited, 132 (80.0%) attended 6-month assessments [24]. From these, 71 (54.0%) participants with obesity (BMI ≥ 97th percentile [23]) completed all the assessments, being included in this exploratory study (CG, *n* = 6; EGI *n* = 30; EGII *n* = 35). Included participants showed a higher age, BMI, and pubertal status than those excluded. No other differences were found between included and excluded participants, including in bone-related parameters (Supplemental Table 1).

### Measurements

Height, body weight, and waist circumference (WC) were measured according to standard procedures. BMI (BMI = weight (kg)/height^2^ (m)) and waist-to-height ratio (WHtR = WC/Height) were calculated. Height z-score and BMI z-score were additionally computed using the World Health Organization (WHO) AnthroPlus calculator.

Body composition was assessed using dual-energy x-ray absorptiometry (DXA), using QDR 12.4 software, according to the National Health and Nutrition Examination Survey (NHANES) protocol [25]. Bone-related key measures variables included areal BMC, BMD, and BMD z-score, derived from total body less head assessment. BMD z-score was determined using DXA reference values from NHANES [26]. Total body fat mass (TBFM), trunk fat mass (Trunk FM), and fat-and-bone-free mass (FBFM) were assessed. Relative body fat mass (BFM) and muscle mass (MM) were computed as percentages of TBFM and FBFM divided by body weight, respectively. Central fat mass (Central FM) was calculated as a percentage of Trunk FM divided by TBFM.

Pubertal status was objectively assessed by a pediatrician according to Tanner stages [27].

MS was defined as the presence of at least two of the following: high resting systolic blood pressure (SBP)/diastolic blood pressure (DBP), high triglycerides (TG), low high-density lipoprotein cholesterol (HDL-C) or high glucose levels. Biochemical analyses were conducted according to standard procedures. Cut-off values for MS components are provided in Supplemental Table 2, according to previously published values [28].

Cardiorespiratory fitness (CRF), i.e., VO2 peak, was measured directly using a gas analyzer during a submaximal exercise test using a cycle ergometer. CRF assessment is extensively described elsewhere [22]. VO_2_ peak (ml/min) was additionally adjusted for body weight (ml/kg/min) and used in the analyses.

PA was assessed with triaxial accelerometers, worn above the right hip, programmed to use a 5-s cycle, during at least one weekend day and two weekdays, except during sleep, bathing, or swimming. Only days with more than 480 min registered, were included in the analysis. Activities between 0 and 149 counts/minute were considered as stationary time [29]; between 150 and 499, light PA (LPA); between 500 and 3999, moderate PA (MPA); and more than 4000 counts/minute vigorous PA (VPA) [30]. The daily average of stationary time, LPA, MPA, VPA, and MVPA (MPA + VPA) were calculated and used in the analysis.

Additional information about the exercise performed, and the frequency of regular (structured or unstructured) PA (times per week), was collected by recall at the time of the PA assessments. Due to high heterogeneity in the PAs performed, PAs were further categorized by exercise type (i.e., aerobic, strength, or combined) based on the main characteristics of the activity.

Dietary intake was assessed using three-day food records (2 weekdays and 1 weekend day) with semi-quantitative scaling (e.g., number of spoons or scoops), according to the 4th edition of *Weight and Food portions *[31]. Each food record underwent independent analysis by three trained nutritionists. Final data were included in the analyses after a group discussion. TEI, protein, carbohydrate (CH), fat, calcium, and vitamin D_3_ daily intake, as well as relative content of saturated fatty acids (SFA), monounsaturated fatty acids (MUFA), polyunsaturated fatty acids (PUFA), and trans fatty acids (Trans FA), were considered as variables of interest. Macronutrient content was calculated by dividing its equivalent in kcal by TEI (kcal/day).

Further information on measures and instruments is available elsewhere [20].

### Statistical analysis

A Per Protocol Analysis (PPA) was performed using IBM SPSS statistics software (version 29.0, IBM, New York, USA). The Shapiro–Wilk test assessed normality for continuous variables. Mean ± SD and median (interquartile range, IR) were calculated for normal and skewed data. Baseline differences between girls and boys were analyzed with independent *t*-tests/Mann–Whitney U tests and Chi-squared for continuous and categorical variables, respectively. Generalized Estimating Equations (GEE), adjusted for age, pubertal status, and group (i.e., trial allocation group), were used to analyze over time changes and sex-by-time interaction in anthropometric, body composition, and bone-related parameters, and also cardiorespiratory fitness, PA, and diet. The associations between changes in these variables were performed with partial correlations controlling for sex, ethnicity, age, pubertal status, group, height, and presence of MS.

The variables significantly associated with over time changes in BMC, BMD, and BMD z-score were further included as independent variables in predictive models, using Linear regressions, adjusting for possible confounders [32].

Additional analyses investigating differences in body composition, diet, and PA behaviors changes by type of exercise were performed using GEE (adjusted for age, sex, pubertal status, and group) and Chi-squared (for categorical variables). Since only one participant performed strength training, data on this participant was gathered to the combined training group. Participants who changed the type of exercise performed during the 6 months were excluded from this specific analysis (*n* = 2, both boys from the combined training group).

A *p*-value of ≤ 0.05 was considered statistically significant.

## Results

Post-hoc power calculation using BMC difference as endpoint, compared to previously published values in a similar population (29.4 ± 5.3) [33], and an alpha of 0.05 yielded a statistical power of 100%.

Seventy-one participants (93.0% Caucasian; 57.7% girls), with a mean age of 15.1 (± 1.6) years and a mean BMI of 34.44 (± 5.00) kg/m2, followed for 6 months, were included. The presence of MS components is presented in Supplemental Table 3. At baseline, the majority of participants presented a normal BMD z-score, yet 2 participants (2.8%) presented a BMD z-score of -2 SD; only 1 participant (1.4%) showed to meet the recommendations for calcium intake (i.e., 960 mg/day) [34]; 6 (8.5%) reported the minimum calcium intake needed to observe a beneficial impact of PA on BMD (i.e., 700 mg/day) [35]; and 19 (26.8%) met PA guidelines (≥ 60 min of MVPA [36]).

Sex differences in height, height z-score, BMI z-score, WC, FBFM (Table 1), TEI (Table 2), and pubertal status (Supplemental Table 3) but not in bone-related variables were found at baseline (Table 1).

**Table 1:** Over time changes in bone-related parameters, anthropometrics, and body composition by sex

	Girls (*n* = 41)		Boys (*n* = 30)		Time	Sex*Time
*Variable*	*Baseline*	*6 months*	*Baseline*	*6 months*	*β* (95% CI)	*β* (95% CI)
Height z-score	0.05 ± 1.04 *	0.03 ± 1.01	0.59 ± 1.15 *	0.47 ± 1.17	**-0.12 (-0.19, -0.04)**	**-0.10 (-0.18, -0.01)**
BMI (kg/m^2^)	33.83 ± 3.90	33.61 ± 4.97	34.33 ± 5.93	34.21 ± 6.44	-0.12 (-0.91, 0.67)	-0.11 (-1.11, 0.89)
BMI z-score	2.79 ± 0.58 *	2.69 ± 0.75	3.26 ± 0.83 *	3.12 ± 0.96	**-0.13 (-0.25, -0.02)**	-0.04 (-0.19, 0.11)
WHtR	0.64 ± 0.06	0.63 ± 0.07	0.66 ± 0.08	0.64 ± 0.09	**-0.02 (-0.03, -0.01)**	-0.02 (-0.03, 0.01)
TBFM (kg)	37.30 ± 8.06	36.87 ± 9.65	38.76 ± 10.62	38.02 ± 12.43	-0.74 (-2.25, 0.77)	-0.32 (-2.18, 1.55)
BFM (%)	42.4 ± 5.6	41.0 ± 5.2	40.1 ± 7.4	37.7 ± 6.0	**-2.10 (-3.10, -1.09)**	**-1.35 (-2.57, -0.13)**
Trunk FM (kg)	17.36 ± 4.74	16.69 ± 4.96	18.01 ± 5.79	16.24 ± 6.55	**-1.77 (-2.67, -0.87)**	**-1.10 (-2.17, -0.02)**
Central FM (%/TBFM)	46.2 ± 4.8	44.9 ± 4.3	46.1 ± 5.2	42.0 ± 5.1	**-4.09 (-5.72, -2.46)**	**-2.83 (-4.68, -0.97)**
FBFM (kg)	46.98 ± 5.71 *	47.60 ± 6.0	53.51 ± 11.43 *	56.91 ± 10.38	**3.40 (1.94, 4.85)**	**-2.78 (-4.32, -1.23)**
MM (%)	53.4 ± 4.1	54.3 ± 5.3	55.4 ± 5.3	57.8 ± 6.0	**2.45 (1.33, 3.57)**	**-1.49 (-2.92, -0.07)**
BMC (kg)	2.04 ± 0.31	2.08 ± 0.31	2.17 ± 0.51	2.31 ± 0.51	**0.14 (0.10, 0.18)**	**-0.10 (-0.15, 0.05)**
BMD (g/m^2^)	1.07 ± 0.09	1.09 ± 0.09	1.05 ± 0.13	1.09 ± 0.14	**0.04 (0.03, 0.05)**	**-0.02 (-0.04, -0.01)**
BMD z-score	0.32 ± 0.93	0.36 ± 0.96	0.10 ± 1.21	0.41 ± 1.12	**0.31 (0.10, 0. 54)**	**-0.26 (-0.49, -0.02)**
	**Girls**		**Boys**		***p***	**Total**
BMC decrease (*n*, %)	11 (27)		2 (6.7)		**.030**	13 (18)
BMD decrease (n, %)	8 (16)		1 (3.3)		**.043**	9 (13)

BFM, body fat mass; BMC, bone mineral content; BMD, bone mineral density; BMI, body mass index; FBFM, fat- and bone-free mass; FM, fat mass; MM, muscle mass; TBFM, total body fat mass; WHtR, waist-height ratio

^*^ Between-group differences at baseline (*p* ≤ .05), analyzed with independent sample *t*-test

Bold $\beta$ s indicate a significant (*p* ≤ .05) time and sex-by-time interaction. Standardized $\beta$ s adjusted for age, pubertal status, and group (trial allocation group)

**Table 2:** Over time changes in diet, physical activity, and cardiorespiratory fitness by sex

		Girls (*n* = 41)			Boys (*n* = 30)	Time	Sex*Time
*Variable*	*Baseline*	*6 months*	*Baseline*	*6 months*		*β* (95% CI)	*β* (95% CI)
TEI (kcal/day)	1139 ± 325 *	1055 ± 325	1346 ± 367 *	1287 ± 339		-59.44 (-151.21, 32.32)	-23.91 (-174.52, 126.58)
Protein (%/TEI)	22.0 (5.5)	26.2 (8.0)	22.2 (6.3)	24.4 (6.3)		1.14 (-0.94, 3.21)	0.12 (-0.01, 0.32)
CH (%/TEI)	43.3 ± 5.3	41.3 ± 7.4	43.3 ± 6.3	42.8 ± 6.1		-1.39 (-3.20, 0.61)	-1.51 (-5.32, 2.29)
Fat (%/TEI)	34.3 ± 5.9	32.0 ± 7.5	33.3 ± 6.0	32.7 ± 6.2		-1.49 (-3.06, 0.75)	-1.71 (-4.89, 1.50)
SFA (%/Total Fat)	32.3 (9.3)	32.9 (10.5)	34.7 (6.2)	34.9 (4.9)		0.77 (-1.01, 2.56)	0.01 (-0.09, 0.11)
MUFA (%/Total Fat)	33.4 ± 7.5	37.8 ± 7.8	33.6 ± 6.7	34.4 ± 6.7		**2.56 (0.59, 4.54)**	3.58 (-0.41, 7.48)
PUFA (%Total Fat)	16.2 (5.7)	14.3 (5.2)	14.4 (5.5)	13.1 (4.5)		-0.89 (-2.25, 0.28)	-0.09 (-0.21, 0.09)
Trans FA (%/Total Fat)	1.9 (2.0)	1.5 (1.5)	2.1 (1.9)	1.7 (0.9)		**-0.57 (-0.85, -0.29)**	-0.01 (-0.29, 0.21)
Calcium Intake (mg/day)	383.0 (236.3)	333.9 (256.6)	430.1 (226.2)	469.2 (427.0)		3.02 (-22.85, 28.89)	**-0.10 (-0.19, -0.11)**
Vitamin D_3_ (µg/day)	1.31 (1.94)	1.95 (1.91)	2.14 (2.00)	2.48 (2.32)		0.21 (-0.13, 0.55)	0.07 (-0.25, 0.39)
Stationary (min/day)	598.1 (180.6)	626.8 (202.1)	633.8 (130.6)	600.5 (111.9)		**-53.64 (-92.26, 15.01)**	**65.52 (11.24, 119.80)**
LPA (min/day)	61.8 (58.7)	55.5 (51.2)	45.6 (78.7)	50.3 (29.4)		-0.45 (-23.54, 22.64)	1.54 (-23.99, 27.08)
MPA (min/day)	36.4 (42.5)	64.3 (73.4)	30.9 (31.9)	76.0 (51.6)		**38.93 (27.23, 50.64)**	**-24.96 (-40.99, -8.93)**
VPA (min/day)	5.8 (8.5)	11.4 (10.8)	5.1 (7.5)	12.2 (14.3)		**7.62 (4.99, 10.24)**	-3.59 (-0.53, 7.72)
MVPA (min/day)	46.5 (47.4)	72.8 (82.8)	35.6 (33.6)	85.4 (51.0)		**46.55 (33.75, 59.35)**	**-28.55 (-46.89, -10.21)**
VO2 peak (ml/kg/min)	20.65 ± 2.44	21.53 ± 3.34	21.76 ± 3.12	23.07 ± 3.95		**1.10 (0.58, 1.62)**	-0.41 (-1.45, 0.63)

CH, carbohydrates; FA, fatty acids; LPA, light physical activity; MPA, moderate physical activity; MUFA, monounsaturated fatty acids; MVPA, moderate-vigorous physical activity; PUFA, polyunsaturated fatty acids; TEI, total energy intake; SFA, saturated fatty acids; VPA, vigorous physical activity

^*^ Between-group differences at baseline (*p* ≤ .05), analyzed with independent sample *t*-test

Bold $\beta$ s indicate a significant (*p* ≤ .05) time and sex-by-time interaction. Standardized $\beta$ s adjusted for age, pubertal status, and group (trial allocation group)

Cross-sectional (baseline) correlations are presented in Supplemental Table 4.

### Changes in bone-related parameters, anthropometrics, and body composition

Overall, participants showed a significant increase in BMC (*β* = 0.14; 95%CI: 0.10, 0.18), BMD (*β* = 0.04; 95%CI: 0.03, 0.05), and BMD z-score (*β* = 0.31; 95%CI: 0.10, 0.54), as well as in FBFM (*β* = 3.40, 95%CI: 1.94, 4.85) and MM (*β* = 2.45, 95%CI: 1.33, 3.57); and a decrease in height z-score (*β* = -0.12; 95%CI: -0.19, -0.04), BMI z-score (*β* = -0.13; 95%CI: -0.25, -0.02;), WHtR (*β* = -0.02; 95%CI: -0.03, -0.01), BFM (*β* = -2.10; 95%CI: -3.10, -1.09), Trunk FM (*β* = -1.77; 95%CI: -2.67, -0.87), and Central FM (*β* = -4.09; 95%CI: -5.72, -2.46). Among all, 13 (18.3%) and 9 (12.7%) participants showed an impairment in BMC and BMD, respectively (Table 1).

A sex-by-time interaction was found, with girls showing a smaller increase in BMC (*β* = -0.10; 95%CI: -0.15, -0.05), BMD (*β* = -0.02; 95%CI: -0.04, -0.01), BMD z-score (*β* = -0.26; 95%CI: -0.49, -0.02), FBFM (*β* = -2.78; 95%CI: -4.32, -1.23), and MM (*β* = -1.49; 95%CI: -2.92, -0.07), compared to boys; as well as a smaller decrease in Height z-score (*β* = -0.10; 95%CI: -0.15, -0.05), BFM (*β* = -1.35; 95%CI: -2.57, -0.13), Trunk FM (*β* = -1.10; 95%CI: -2.17, -0.02), Central FM (*β* = -2.83; 95%CI: -4.68, -0.97). More girls showed an impairment in BMC and BMD than boys (Table 1).

### Changes in diet, physical activity, and cardiorespiratory fitness

Participants showed an significant increase in MUFA (*β* = 2.56; 95%CI:, 0.59, 4.54), MPA (*β* = 38.93; 95%CI: 27.23, 50.64), VPA (*β* = 7.62; 95%CI: 4.99, 10.24), MVPA (*β* = 46.55; 95%CI: 33.75, 59.35), and CRF (*β* = 1.10; 95%CI: 0.58, 1.62); as well as a decrease in Trans FA (*β* = -0.57; 95%CI:, -0.85, -0.29) and stationary time (*β* = 53.64; 95%CI:, 92.26, 15.01) (Table 2).

A sex-by-time interaction was found, with girls showing a higher increase in time spent stationary, compared to a decrease in boys (*β* = 65.52; 95%CI: 11.24, 119.80; *p* = 0.020); and a smaller increase in MPA (*β* = -24.96; 95%CI: -40.99, -8.93) and MVPA (*β* = -28.55; 95%CI: -46.89, -10.21) (Table 2).

### Association between changes in bone parameters, anthropometrics, diet, and physical activity

BMC changes showed a significant correlation with WHtR (*r* = -0.322) and VO_2_ peak (*r* = 0.306) (Table 3). Changes in BMD were positively correlated with changes in CH intake (*r* = 0.432). BMD z-score changes were negatively correlated with changes in BMI z-score (*r* = -0.409), WHtR (*r* = -0.374), BFM% (*r* = -0.353), LPA (*r* = -0.345), and protein content (%) (*r* = -0.462); and positively with MM% (*r* = 0.412), VO_2_ peak (*r* = 0.436), and CH content (*r* = 0.317). No correlations were found between BMC/BMD/BMD z-score and changes in SFA, MUFA, PUFA, or Trans FA intake (data not shown).

**Table 3:** Partial correlations between changes in bone-related parameters, anthropometrics, diet, and physical activity

Δ	BMI z-score	WHtR	BFM (%)	Trunk FM (kg)	MM (%)	Stationary (min/day)	LPA (min/day)	MPA (min/day)	VPA (min/day)	MVPA (min/day)	VO_2_ peak (ml/kg/min)	TEI (kcal/day)	Protein (%TEI)	CH (%TEI)	Fat (%TEI)	Calcium (g)	Vitamin D (µg)	BMC (g)	BMD (g/m2)	BMD z-score
						(1)	(1)	(1)	(1)	(1)			(1)			(1)				
BMI z	1																			
WHtR	**.850 §**	1																		
BFM	**.371 †**	.366 *	1																	
TrunkFM	**.629 §**	**.502 §**	**.805 §**	1																
MM	**-.668 §**	**-.514 §**	**-.557 §**	-.217	1															
Stationary (1)	.010	-.049	.024	.072	-.027	1														
LPA (1)	-.019	-.037	.195	.256	.082	.096	1													
MPA (1)	**-.403 †**	-.319 *	**-.471 §**	-.206	**.475 §**	-.126	-.083	1												
VPA (1)	-.205	-.116	**-.426 §**	-.239	**.428 †**	-.197	-.035	**.449 §**	1											
MVPA (1)	**-.398 †**	-.303 *	**-.514 §**	-.238	**.517 §**	-.159	-.080	**.973 §**	**.643 §**	1										
VO_2_ peak	**-.706 §**	**-.556 §**	**-.470 §**	**-.392 †**	**.585 §**	-.123	-.134	**.426 †**	**.418 †**	**.473 §**	1									
TEI	.069	.055	.128	.100	-.015	-.004	.017	-.007	-.132	-.040	-.209	1								
Protein (1)	-.083	-.151	.012	-.051	-.070	.124	.307 *	-.028	.032	-.016	-.082	**-.506 §**	1							
CH	-.093	.095	-.016	.107	-.010	-.154	-.203	-.059	-.043	-.062	-.097	.197	**-.516 §**	1						
Fat	-.177	-.138	.012	.064	.078	.043	-.083	.090	.014	-.081	.182	.282	**-.425 †**	**-.556 §**	1					
Calcium (1)	.068	.131	.086	-.079	-.054	.064	.068	.070	-.152	.020	-.126	**.429 §**	.098	-.102	.013	1				
Vitamin D	-.050	-.198	.020	.039	.072	-.005	.126	.141	.077	.141	-.073	.201	.076	-.316 *	.259	**.371 †**	1			
BMC	-.209	-.322 *	.014	-.038	.039	-.163	-.268	-.017	-.028	-.021	.306 *	-.053	-.024	-.226	-.215	-.081	-.237	1		
BMD	-.086	-.144	-.145	-.074	.234	-.258	-.149	-.166	.022	-.136	.098	.047	-.235	**.432 †**	-.228	-.065	-.194	**.475§**	1	
BMD z	**-.409 †**	**-.374 †**	-.353 *	-.177	**.412 †**	-.197	-.345 *	.145	.194	.174	**.436 †**	.002	**-.462 §**	.317 *	.113	-.051	-.093	.331 *	**.436 §**	1

Parametric and non-parametric ^(1)^ correlations controlling for sex, ethnicity, age, pubertal status, group (trial allocation group), height, and presence of metabolic syndrome

BFM, body fat mass; BMC, bone mineral content; BMD, bone mineral density; BMI, body mass index; CH, carbohydrates; LPA, light physical activity; MM, muscle mass; MPA, moderate physical activity; MVPA, moderate-vigorous physical activity; TEI, total energy intake; Trunk FM, trunk fat mass; VPA, vigorous physical activity; WHtR, waist-height ratio

^*^ *p*-value < .05; † *p*-value < .01; § *p*-value < .001

### Bone mineral content and bone mineral density variance

BMC variance was not explained by LPA, protein, or CH changes (Table 4).

**Table 4:** Regression models with over time variation in bone mineral content, bone mineral density, and bone mineral density z-score as dependent variables

	Model 1			Model 2			Model 3			Model 4		
*Δ BMC (kg)*	*β*	95% CI	*p*	*β*	95% CI	*p*	*β*	95% CI	*p*	*β*	95% CI	*p*
Δ LPA (min/day)	-0.10	-0.78, 0.38	.497	-0.02	-0.54, 0.44	.844	-0.10	-0.65, 0.23	.343	-0.11	-0.66, 0.21	.310
Δ Protein (%/TEI)	-0.07	-5.29, 3.35	.654	0.04	-3.32, 4.46	.771	0.14	-3.27, 3.67	.908	-0.03	-4.37, 3.38	.821
Δ CH (%/TEI)	-0.06	-4.60, 3.09	.696	-0.14	-5.04, 1.51	.284	-0.12	-4.51, 1.42	.300	-0.08	-4.13, 2.04	.499
*Δ BMD (g/m*^*2*^*)*												
Δ LPA	-0.13	0.00, 0.00	.389	-0.09	0.00, 0.00	.513	-0.09	0.00, 0.00	.536	-0.08	0.00, 0.00	.572
Δ Protein (%/TEI)	-0.40	-0.00, -0.01	**.005**	-0.37	-0.03, 0.00	.**011**	-0.38	-0.03, 0.00	**.010**	-0.34	-0.03, 0.00	**.040**
Δ CH (%/TEI)	0.40	0.00, 0.03	**.006**	0.38	0.00, 0.03	**.007**	0.43	0.01, 0.03	**.002**	0.42	0.01, 0.03	**.004**
*Δ BMD z-score*												
Δ LPA	-0.18	-0.01, 0.01	.204	-0.15	-0.01, 0.01	.288	-0.11	-0.03, 0.01	.428	-0.10	-0.03, 0.01	.463
Δ Protein (%/TEI)	-0.31	-0.04, -0.00	**.015**	-0.30	-0.04, -0.00	**.022**	-0.30	-0.04, -0.00	**.018**	-0.41	-0.04, -0.01	**.004**
Δ CH (%/TEI)	0.24	-0.00, 0.03	.081	0.21	-0.00, 0.03	.119	0.29	0.01, 0.03	**.024**	0.29	0.01, 0.03	**.028**

BMC, bone mineral content; BMD, bone mineral density; CH, carbohydrates; LPA, light physical activity; TEI, total energy intake

Model 1: adjusted for baseline LPA, Protein or CH levels, respectively; Model 2: Model 1 + sex, age, pubertal status, group (trial allocation group), baseline muscle mass (%), and metabolic syndrome; Model 3: Model 2 + exercise type and VO_2_ peak; Model 4: Model 3 + TEI, protein and CH intake for LPA, and TEI for Protein and CH

BMD variance was explained by changes in protein and CH content but not by LPA. Changes in protein content were negatively associated with BMD even when adjusting for all the confounders (Model 4: *β* = -0.34, 95%CI: -0.03 to 0.00). Conversely, changes in CH content were positively associated with BMD (Model 4: *β* = 0.42, 95%CI: 0.01 to 0.03).

Similar results were found regarding BMD z-score variance. Adjusting for all the confounding variables (Model 4), changes in protein and CH content were shown to negatively and positively predict BMD z-score variance, respectively (*β* = -0.41, 95%CI: -0.04, -0.01; *β* = 0.29, 95%CI: 0.01, 0.03) (Table 4).

### Changes in body composition, bone parameters, diet, and physical activity behaviors by exercise type

Participants exposed to combined training showed a higher decrease in BMI z-score (*β* = -0.21, 95%CI: -0.35 to -0.07), WHtR (*β* = -0.02, 95%CI: -0.04 to -0.01), Trunk FM (*β* = -1.75, 95%CI: -2.77 to -0.73), and stationary time (*β* = -65,17, 95%CI: -122.79 to -7.55); and a higher increase in MM (*β* = 1.95, 95%CI: 0.53 to 3.39), BMC (*β* = 0.09, 95%CI: 0.05 to 0.13), MVPA (*β* = 35.58, 95%CI: 16.67 to 54.48), and VO_2_ peak (*β* = 1.71, 95%CI: 0.76 to 2.66) compared to those exposed to aerobic exercise. No differences were found in BMD or BMD z-score between aerobic and combined exercise training groups (Supplemental Table 4).

## Discussion

This study sought to explore the influence of diet and PA changes on BMC and BMD alterations in adolescents with obesity under a weight loss program, which assumes particular importance as this population may be at a higher risk of early development of osteopenia/osteoporosis, not only due to an adverse effect of obesity and MS on bone metabolism [9, 12], but also due to an inadequate diet, including low calcium intake [37], and low levels of PA [38] (usually observed in this population).

In fact, despite an overall 6-month increase in BMC, BMD and BMD z-score, 2 participants (2.8%) presented an impairment in BMD z-score of -2 SD at baseline and 9 (12.7%) showed an over time decrease in BMD. Changes in MM (%) were positively associated with BMD z-score, and changes in WHtR were inversely associated with both BMC and BMD z-score, suggesting that abdominal adiposity and proinflammatory adipokines have a deleterious effect on bone health, as previously reported by other authors [11, 39, 40].

Regarding the potential influence of diet on bone-related parameters, it is noteworthy that only macronutrient intake was associated with BMD/BMD z-score. Specifically, increases in CH content were associated with increases in BMD and BMD z-score; and increases in protein content were associated with decreases in BMD and BMD z-score. These associations persisted after adjusting for confounders. Though unexpected, these findings may be associated with a high animal protein consumption and (relatively) low CH intake leading to a possible hypercalcinuria, increasing urinary calcium excretion in these participants [41]. Others have suggested a deleterious effect of high CH intake on calcium metabolism, yet this effect depends on food sources [42]. High CH intake from natural food sources, rich in complex digestible CH, also providing dietary fiber, phytic acid, and oxalic acid – elements that can bind to calcium, may decrease gastrointestinal calcium absorption and bioavailability [42]. However, this mechanism may not apply to our participants, whose CH intake primarily comes from processed sources, poor in complex CH [1].

No associations were found between changes in PA and bone-related parameters when controlling for changes in body composition and diet. It has been suggested that higher PA intensities are positively associated with bone health in adolescents [43]. The lack of association between MVPA and bone-related parameters in this study may be explained by the fact that the impact of PA on bone metabolism may depend on calcium intake and availability and/or exercise characteristics (i.e., type of exercise). Indeed, only 6 (8.5%) participants reported the minimum intake suggested as needed to observe a beneficial impact of PA on BMD [35], with longitudinal data showing a significant overall increase in MVPA but not in calcium intake. In turn, a time-by-type of exercise (i.e., aerobic vs. combined) effect was observed on adiposity and bone parameters, with combined training showing better outcomes in BMI z-score, WHtR, Trunk FM, MM, and BMC compared to the aerobic one. This evidence aligns with the literature, especially concerning aerobic non-weight-bearing activities, such as swimming [44]. Combined training, particularly resistance exercise, exerts an increased mechanical load on bone, improving bone strength, compared to aerobic non-weight-bearing activities [44, 45].

The limited number of participants and the use of self-reported diet data are the two major limitations of the present study. As it has been previously suggested, adolescents with obesity tend to underreport food intake [46], which could lead to an underestimation of calcium intake. Yet, a possible underestimation of calcium intake would not be significative in our sample since adolescents with obesity tend to underreport energy-dense/low-quality foods and drinks, poor in calcium [1, 46]. The difference found between included and excluded participants regarding variables that are known to be associated with bone development (i.e., age, BMI, and pubertal status) can also be considered as a limitation, which was addressed by controlling data analysis for numerous confounding factors, including the ones mentioned, avoiding interpretation bias. Although the aim of the present study was not to investigate in depth the impact of exercise (but daily PA) on bone-related parameters, the high heterogeneity in the PAs reported by the participants did not allow further analysis, conditioning, for example, its categorization in just type of exercise (aerobic vs. combined), which may also be considered as a limitation.

Despite the acknowledged limitations, this study adds to the previous literature by exploring the influence of diet and PA changes on BMC/BMD of adolescents with obesity under a weight loss program using objectively measured PA and controlling for important potential confounding factors.

This study highlights the complex interplay between adiposity, diet, PA, and bone health in adolescents. Improvements in adiposity, MM, and higher CH diet showed associations with BMD/BMD z-score enhancement. Moreover, while exercise type (i.e., combined training vs. aerobic) may moderate the impact of PA on bone health, low calcium intake may compromise the possible effect of exercise on bone health, emphasizing the need for nutritional monitoring. Comprehensive monitoring of diet and PA behaviors, and not only energy balance, is essential for effective intervention strategies in this population.

### Supplementary Information

Below is the link to the electronic supplementary material.

Supplementary file1 (DOCX 124 KB)

## Data Availability

The data that support the findings of this study are available from the corresponding author, AVS, upon reasonable request.

## Abbreviations

BFM: Body fat mass
BMC: Bone mineral content
BMD: Bone mineral density
BMI: Body mass index
CH: Carbohydrate
FBFM: Fat-and-bone-free mass
MVPA: Moderate-vigorous physical activity
PA: Physical activity
TBFM: Total body fat mass
TEI: Total energy intake
WC: Waist circumference
WHtR: Waist-to-height ratio
