# Supplementary material for: Dietary content and combined training, but not daily physical activity, are associated with 6-month bone mineral changes in adolescents with obesity: A Secondary analysis of the PAC-MAnO trial
Source: Eur J Pediatr. 2024 Jun 27;183(9):3969–78. doi: 10.1007/s00431-024-05659-4 (PMC11322498; doi:10.1007/s00431-024-05659-4)
Supplement: Supplementary file 1 — Supplementary file1 (DOCX 124 KB) [file 431_2024_5659_MOESM1_ESM.docx]

**Supplemental Table 1.** Differences in anthropometrics and body composition between participants included and those excluded from analysis.

| ***Outcome*** |  | **Included** |  | **Excluded** |  |  |
| --- | --- | --- | --- | --- | --- | --- |
|  |  |  | **Mean ± SD** | |  | ***p*** |
| Age (years) |  | 15.1 ± 1.6 | (*n* = 71) | 14.2 ± 1.8 | (*n* = 61) | **.019 ^a^** |
| Weight (kg) |  | 91.3 (20.5) * | (*n* = 71) | 86.3 (15.2) * | (*n* = 61) | .092 ^b^ |
| Height (cm) |  | 164.4 ± 7.9 | (*n* = 71) | 163.7 ± 8.7 | (*n* = 61) | .745 ^a^ |
| Height z-score |  | 0.28 ± 1.11 | (*n* = 71) | 0.45 ± 1.13 | (*n* = 61) | .376 ^a^ |
| BMI (kg/m^2^) |  | 34.02 (7.00) * | (*n* = 71) | 31.38 (7.16) * | (*n* = 61) | **.035 ^b^** |
| BMI z-score |  | 2.93 (0.91) * | (*n* = 71) | 2.70 (0.90) * | (*n* = 61) | .239 ^b^ |
| WC (cm) |  | 105.4 (15.0) * | (*n* = 71) | 101.7 (14.6) * | (*n* = 56) | .370 ^b^ |
| BFM (%) |  | 40.9 ± 5.0 | (*n* = 71) | 40.3 ± 5.8 | (*n* = 15) | . 675 ^a^ |
| Central FM (%/TBFM) |  | 46.1 ± 4.9 | (*n* = 71) | 45.5 ± 4.8 | (*n* = 15) | .647 ^a^ |
| MM (%) |  | 54.2 ± 4.7 | (*n* = 71) | 54.7 ± 5.6 | (*n* = 15) | .719 ^a^ |
| BMC (kg) |  | 2.11 ± 0.43 | (*n* = 71) | 2.23 ± 0.45 | (*n* = 15) | .051 ^a^ |
| BMD (g/m^2^) |  | 1.06 ± 0.11 | (*n* = 71) | 1.08 ± 0.08 | (*n* = 15) | .504 ^a^ |
| BMD z-score |  | 0.20 ± 1.09 | (*n* = 71) | 0.47 ± 0.89 | (*n* = 15) | .235 ^a^ |
|  |  |  |  |  |  |  |
|  |  |  | ***n* (%)** | |  | ***p*** |
| Ethnicity (Caucasian) |  | 66 (93) | (*n* = 71) | 53 (87) | (*n* = 61) | .122 ^c^ |
| Sex (Girls) |  | 41 (58) | (*n* =71) | 31 (51) | (*n* = 61) | .363 ^c^ |
| Tanner stage | II | 6 (8.5) | (*n* = 71) | 9 (14) | (*n* = 61) | **.026 ^c^** |
|  | III | 10 (14) |  | 10 (16) |  |  |
|  | IV | 11 (16) |  | 21 (33) |  |  |
|  | V | 44 (62) |  | 24 (38) |  |  |

BFM, body fat mass; BMC, bone mineral content; BMD, bone mineral density; BMI, body mass index; MM, muscle mass; WC, waist circumference.

^a, b, c^ Between-group differences analyzed with Independent-sample *t*-test, Mann-Whitney U Test, and Chi-squared, respectively.

* Median (Interquartile range) is presented for non-normal distributed variables.

**Supplemental Table 2.** Cut-off values used to determine the presence of metabolic syndrome and related components (according to previously published values)[28].

|  |  |  |  | Age | | | | | | |  |
| --- | --- | --- | --- | --- | --- | --- | --- | --- | --- | --- | --- |
|  |  |  |  |  |  |  |  |  |  |  |  |
|  |  |  |  | **12** | **13** | **14** | **15** | **16** | **17** | **18** |  |
| **Metabolic Syndrome** |  |  |  |  |  |  |  |  |  |  |  |
|  | **SBP** | (mmHg) | Both | ≥ 121 | ≥ 123 | ≥ 125 | ≥ 126 | ≥ 128 | ≥ 128 | ≥ 129 |  |
|  |  |  |  |  |  |  |  |  |  |  |  |
|  |  |  |  |  |  |  |  |  |  |  |  |
|  | **DBP** | (mmHg) | Girls | ≥ 80 | ≥ 82 | ≥ 83 | ≥ 84 | ≥ 84 | ≥ 85 | ≥ 85 |  |
|  |  |  | Boys | ≥ 76 | ≥ 78 | ≥ 79 | ≥ 81 | ≥ 82 | ≥ 83 | ≥ 84 |  |
|  |  |  |  |  |  |  |  |  |  |  |  |
|  | **TG** | (mg/dl) | Girls | ≥ 141 | ≥ 135 | ≥ 129 | ≥ 127 | ≥ 129 | ≥ 135 | ≥ 142 |  |
|  |  |  | Boys | ≥ 127 | ≥ 130 | ≥ 134 | ≥ 138 | ≥ 140 | ≥ 143 | ≥ 146 |  |
|  |  |  |  |  |  |  |  |  |  |  |  |
|  | **HDL-C** | (mg/dl) | Girls | ≤ 44 | ≤ 43 | ≤ 41 | ≤ 40 | ≤ 40 | ≤ 40 | ≤ 40 |  |
|  |  |  | Boys | ≤ 48 | ≤ 48 | ≤ 49 | ≤ 49 | ≤ 49 | ≤ 49 | ≤ 50 |  |
|  | **Glucose** | (mg/dl) | Both | ≥ 101 | | | | | | |  |
|  |  |  |  |  |  |  |  |  |  |  |  |
|  |  |  |  |  |  |  |  |  |  |  |  |
|  |  |  |  |  |  |  |  |  |  |  |  |

DBP, diastolic blood pressure; HDL-C, high-density lipoprotein cholesterol; SBP, systolic blood pressure; TG, triglycerides.

**Supplemental Table 3.** Participants’ baseline characteristics.

| ***Outcome*** |  | **Girls (*n*=41)** | **Boys (*n*=30)** |  | **Total (*n*=71)** |
| --- | --- | --- | --- | --- | --- |
|  |  | **Mean ± SD** | | ***p*** | **Mean ± SD** |
| Age (years) |  | 15.3 ± 1.3 | 14.5 ± 2.0 | .062 ^a^ | 15.1 ± 1.6 |
| Weight (kg) |  | 88.6 ± 12.9 | 97.2 ± 21.8 | .059 ^a^ | 93.7 ± 18.9 |
| Height (cm) |  | 161.6 ± 6.4 | 167.7 ± 8.5 | **.001 ^a^** | 164.4 ± 7.9 |
| Height z-score |  | 0.05 ± 1.04 | 0.59 ± 1.15 | **.044 ^a^** | 0.28 ± 1.11 |
| BMI (kg/m^2^) |  | 33.83 ± 3.90 | 34.33 ± 5.93 | .671 ^a^ | 34.44 ± 5.00 |
| BMI z-score |  | 2.79 ± 0.58 | 3.26 ± 0.83 | **.007 ^a^** | 3.03 ± 0.78 |
| WC (cm) |  | 103.3 ± 9.8 | 110.4 ± 14.4 | **.016 ^a^** | 107.4 ± 13.4 |
| BMC (kg) |  | 2.04 ± 0.31 | 2.17 ± 0.51 | .173 ^a^ | 2.11 ± 0.43 |
| BMD (g/m^2^) |  | 1.07 ± 0.09 | 1.05 ± 0.13 | .289 ^a^ | 1.06 ± 0.11 |
| BMD z-score |  | 0.32 ± 0.93 | 0.10 ± 1.21 | .423 ^a^ | 0.20 ± 1.09 |
|  |  |  |  |  |  |
|  |  | ***n* (%)** | | ***p*** | ***n* (%)** |
| Ethnicity (Caucasian) |  | 38 (93) | 28 (93) | .916 ^b^ | 66 (93) |
| Tanner stage | II | 0 (0.0) | 6 (20) | **<.001 ^b^** | 6 (8.5) |
|  | III | 2 (4.9) | 8 (27) |  | 10 (14) |
|  | IV | 5 (12) | 6 (20) |  | 11 (16) |
|  | V | 34 (83) | 10 (33) |  | 44 (62) |
| High BP ^(1)^ |  | 8 (20) | 9 (30) | .306 ^b^ | 17 (24) |
| High glycemia ^(1)^ |  | 1 (2.4) | 1 (3.3) | .836 ^b^ | 2 (2.8) |
| Low HDL-C ^(1)^ |  | 8 (20) | 9 (30) | .334 ^b^ | 17 (24) |
| High Triglycerides^)^ |  | 0 (0.0) | 0 (0.0) | - | 0 (0.0) |
| Metabolic Syndrome ^(1)^ |  | 1 (2.4) | 3 (10) | .172 ^b^ | 4 (5.6) |

BFM, body fat mass; BMC, bone mineral content; BMD, bone mineral density; BMI, body mass index; BP, blood pressure; WC, waist circumference.

^(1)^ The cut-off values for metabolic syndrome components are presented in Supplemental Table 2.

^a, b^ Between-group differences analyzed with Independent-sample *t*-test and Chi-squared, respectively.

**Supplemental Table 4.** Partial correlations between bone-related parameters, anthropometrics, diet, and physical activity at baseline.

|  | BMI  z-score | WHtR | BFM (%) | Trunk FM (kg) | MM (%) | Stationary (min/day) | LPA (min/day) | MPA (min/day) | VPA (min/day) | MVPA (min/day) | | VO_2_ peak  (ml/kg/min) | TEI (kcal/day) | Protein (%TEI) | CH (%TEI) | Fat (%TEI) | Calcium (g) | Vitamin D (µg) | BMC (g) | BMD (g/m2) | BMD z-score |
| --- | --- | --- | --- | --- | --- | --- | --- | --- | --- | --- | --- | --- | --- | --- | --- | --- | --- | --- | --- | --- | --- |
|  |  |  |  |  |  | ^(1)^ | ^(1)^ | ^(1)^ | ^(1)^ | ^(1)^ |  | |  | ^(1)^ |  |  | ^(1)^ |  |  |  |  |
| BMI z | 1 |  |  |  |  |  |  |  |  |  |  | |  |  |  |  |  |  |  |  |  |
| WHtR | **.871 §** | 1 |  |  |  |  |  |  |  |  |  | |  |  |  |  |  |  |  |  |  |
| BFM | **.587 §** | **.604 §** | 1 |  |  |  |  |  |  |  |  | |  |  |  |  |  |  |  |  |  |
| TrunkFM | **.805 §** | **.838 §** | **.805 §** | 1 |  |  |  |  |  |  |  | |  |  |  |  |  |  |  |  |  |
| MM | **-.666 §** | **-.680 §** | **-.939 §** | **-.808 §** | 1 |  |  |  |  |  |  | |  |  |  |  |  |  |  |  |  |
| Stationary^(1)^ | .043 | .072 | .003 | -.009 | .008 | 1 |  |  |  |  |  | |  |  |  |  |  |  |  |  |  |
| LPA ^(1)^ | .032 | -.076 | -.086 | .091 | .062 | -.095 | 1 |  |  |  |  | |  |  |  |  |  |  |  |  |  |
| MPA ^(1)^ | -.087 | -.147 | -.134 | -.155 | .195 | **-.258 *** | -.093 | 1 |  |  |  | |  |  |  |  |  |  |  |  |  |
| VPA ^(1)^ | -.165 | -.184 | **-.308 *** | **-.251 *** | **.303 *** | -.165 | .133 | **.617 §** | 1 |  |  | |  |  |  |  |  |  |  |  |  |
| MVPA (1) | -.108 | -.164 | -.179 | -.185 | .230 | **-.256 *** | -.053 | **.987 §** | **.736 §** | 1 |  | |  |  |  |  |  |  |  |  |  |
| VO_2_ peak | **-.680 §** | **-.663 §** | **-.772 §** | **-.743 §** | **.754 §** | -.097 | -.014 | .187 | **.287 *** | .220 | 1 | |  |  |  |  |  |  |  |  |  |
| TEI | .025 | -.022 | -.016 | -.029 | .020 | .092 | .041 | -.046 | .021 | -.035 | .049 | | 1 |  |  |  |  |  |  |  |  |
| Protein ^(1)^ | -.088 | .015 | .046 | .023 | -.042 | .023 | .206 | .010 | -.120 | -.016 | -.038 | | **-.448 §** | 1 |  |  |  |  |  |  |  |
| CH | -.052 | -.159 | -.233 | -.190 | .162 | .089 | -.246 | .076 | -.063 | .053 | .241 | | **.288 *** | **-.344 †** | 1 |  |  |  |  |  |  |
| Fat | .118 | .139 | .186 | .163 | -.121 | -.102 | .074 | -.080 | .154 | -.038 | -.200 | | .074 | **-.450 §** | **-.684 †** | 1 |  |  |  |  |  |
| Calcium ^(1)^ | -.021 | -.082 | -.116 | -.055 | .094 | .054 | .212 | -.052 | .069 | -.031 | .208 | | **.569 §** | **-.325 *** | .114 | .144 | 1 |  |  |  |  |
| Vitamin D | -.158 | -.165 | -.158 | -.186 | .044 | .004 | .089 | .001 | .079 | .017 | .092 | | .165 | .016 | .190 | -.187 | .221 | 1 |  |  |  |
| BMC | .239 | .159 | -.172 | .074 | .035 | -.084 | -.060 | -.141 | -.119 | -.146 | .165 | | -.014 | -.165 | .044 | .086 | .144 | .094 | 1 |  |  |
| BMD | .151 | .053 | **-.338 †** | -.063 | .223 | -.054 | .167 | -.164 | .070 | -.127 | .187 | | .039 | -.225 | .045 | .132 | .239 | .036 | .827 § | 1 |  |
| BMD z | .059 | -.045 | **-.309 *** | -.119 | .221 | .028 | .062 | -.127 | .071 | -.094 | **.298 *** | | .134 | **-.406 †** | .195 | .133 | **.290 *** | .064 | .770 § | .889 § | 1 |
|  |  |  |  |  |  |  |  |  |  |  |  | |  |  |  |  |  |  |  |  |  |

Parametric and non-parametric ^(1)^ correlations controlling for sex, ethnicity, age, pubertal status, height, and presence of metabolic syndrome.

BFM, body fat mass; BMC, bone mineral content; BMD, bone mineral density; BMI, body mass index; CH, carbohydrates; LPA, light physical activity; MM, muscle mass; MPA, moderate physical activity; MVPA, moderate-vigorous physical activity; TEI, total energy intake; Trunk FM, trunk fat mass; VPA, vigorous physical activity; WHtR, waist-height ratio.

* *p*-value <.05; † *p*-value <.01; § *p*-value <.001.

**Supplemental Table 5.** Over time changes in body composition, diet, and physical activity by type of exercise (based on the structured physical activity reported).

|  |  | **Aerobic (*n*= 29)** | |  | **Combined (*n*= 40)** | |  | **Time** | **Ex. Type * Time** |
| --- | --- | --- | --- | --- | --- | --- | --- | --- | --- |
| ***Variable*** |  | ***Baseline*** | ***6 months*** |  | ***Baseline*** | ***6 months*** |  | ***β* (95% CI)** | ***β* (95% CI)** |
| BMI z-score |  | 2.99 ± 0.79 | 3.00 ± 0.96 |  | 2.98 ± 0.70 | 2.79 ± 0.79 |  | **-0.23 (-0.31, -0.14)** | **-0.21 (-0.35, -0.07)** |
| WHtR |  | 0.65 ± 0.07 | 0.65 ± 0.08 |  | 0.64 ± 0.07 | 0.63 ± 0.08 |  | **-0.03 (-0.03, -0.02)** | **-0.02 (-0.04, -0.01)** |
| BFM (%) |  | 42.2 ± 4.2 | 41.5 ± 4.9 |  | 40.0 ± 5.3 | 38.3 ± 6.0 |  | **-1.86 (-2.67, -1.04)** | -0.99 (-2.24, 0.24) |
| Trunk FM (kg) |  | 17.89 ± 5.27 | 17.79 ± 5.89 |  | 17.46 ± 5.17 | 15.61 ± 5.36 |  | **-1.87 (-2.62, -1.11)** | **-1.75 (-2.77, -0.73)** |
| MM (%) |  | 53.2 ± 4.2 | 53.6 ± 5.1 |  | 54.9 ± 4.9 | 57.3 ± 5.8 |  | **2.56 (1.57, 3.55)** | **1.95 (0.53, 3.39)** |
| BMC (kg) |  | 2.02 ± 0.35 | 2.05 ± 0.33 |  | 2.14 ± 0.44 | 2.26 ± 0.45 |  | **0.12 (0.09, 0.16)** | **0.09 (0.05, 0.13)** |
| BMD (g/m^2^) |  | 1.06 ± 0.08 | 1.07 ± 0.09 |  | 1.07 ± 0.12 | 1.10 ± 0.13 |  | **0.03 (0.02, 0.04)** | 0.01 (-0.00, 0.03) |
| BMD z-score | | 0.21 ± 0.82 | 0.28 ± 0.86 |  | 0.24 ± 1.18 | 0.44 ± 1.11 |  | **0.22 (0.05, 0.39)** | 0.13 (-0.10, 0.36) |
| TEI (kcal/day) |  | 1250 ± 328 | 1158 ± 251 |  | 1222 ± 378 | 1162 ± 327 |  | -74.17 (-165.53, 17.19) | 31.30 (-133.62 , 196.22) |
| Protein (%/TEI) |  | 22.2 ± 3.7 | 24.8 ± 5.7 |  | 23.2 ± 5.3 | 26.3 ± 6.3 |  | **3.15 (0.92, 5.38)** | 0.53 (-3.04, 4.10) |
| CH (%/TEI) |  | 43.7 ± 6.5 | 41.7 ± 6.9 |  | 43.1 ± 5.3 | 42.1 ± 6.9 |  | -1.28 (-3.79, 1.23) | 0.99 (-3.08, 5.06) |
| Fat (%/TEI) |  | 34.0 ± 6.1 | 33.5 ± 6.7 |  | 33.7 ± 5.9 | 31.6 ± 7.0 |  | -1.87 (-3.97, 0.23) | -1.52 (-4.89, 1.85) |
| Calcium Intake (mg/day) |  | 415.8 ± 160.3 | 393.6 ± 171.8 |  | 417 ± 2013.8 | 432.8 ± 223.7 |  | 14.05 (-16.67, 44.78) | 38.03 (-19.10, 95.17) |
| Stationary (min/day) |  | 606.8 ± 112.8 | 629.5 ± 103.7 |  | 613.1 ± 134.5 | 570.7 ± 157.2 |  | -30.75 (-68.70, 7.21) | **-65.17 (-122.79, -7.55)** |
| LPA (min/day) |  | 61.8 (105.0) | 64.5 (70.2) |  | 52.4 (59.8) | 53.2 (37.7) |  | 7.11 (-10.18, 24.39) | 12.72 (-11.48, 36.92) |
| MVPA (min/day) |  | 34.9 (34.8) | 40.8 (51.3) |  | 42.3 (47.2) | 101.3 (170.3) |  | **45.71 (31.28, 60.13)** | **35.58 (16.67, 54.48)** |
| VO2 peak (ml/kg/min) |  | 20.69 ± 0.41 | 21.05 ± 0.64 |  | 21.44 ± 0.48 | 23.37 ± 0.55 |  | **1.93 (1.32, 2.55)** | **1.71 (0.76, 2.66)** |
|  |  | **Aerobic** | |  | **Combined** | | ***p*** | **Total** | |
| Age (years) |  | 15.0 ± 1.8 | |  | 14.9 ± 1.7 | | .740 | 13 (18) | |
| Sex (Girls n, %) |  | 23 (79) | |  | 18 (45) | | **.008** | 41 (59) | |
| Frequency (days/week) |  | 2.5 ± 0.9 | |  | 2.4 ± 0.8 | | .796 | 2.4 ± 0.9 | |

BFM, body fat mass; BMC, bone mineral content; BMD, bone mineral density; BMI, body mass index; CH, carbohydrates; FM, fat mass; LPA, light physical activity; MM, muscle mass; MVPA, moderate-physical activity; TEI, total energy intake; WHtR, waist-height ratio.

No statistically significant differences were found (*p* >.05) at baseline between aerobic and combined exercise groups.

Bold $\beta$s indicate a significant (*p* ≤.05) time and group-by-time interaction. Standardized $\beta$s adjusted for age, sex, pubertal status, and group (trial allocation group).


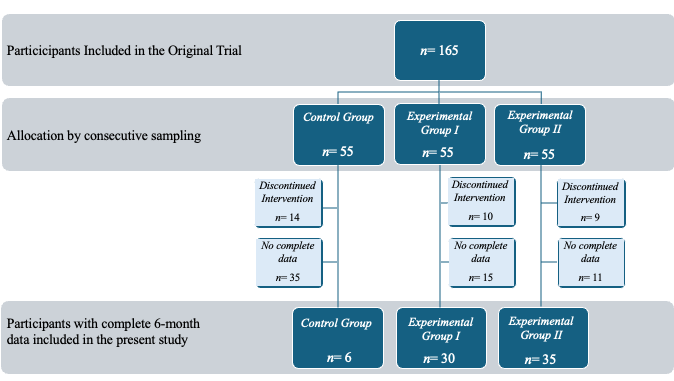


**Supplemental Figure 1.** Study flow diagram.
